# Substantial Contribution of Submicroscopical *Plasmodium falciparum* Gametocyte Carriage to the Infectious Reservoir in an Area of Seasonal Transmission

**DOI:** 10.1371/journal.pone.0008410

**Published:** 2009-12-22

**Authors:** André Lin Ouédraogo, Teun Bousema, Petra Schneider, Sake J. de Vlas, Edith Ilboudo-Sanogo, Nadine Cuzin-Ouattara, Issa Nébié, Will Roeffen, Jan Peter Verhave, Adrian J. F. Luty, Robert Sauerwein

**Affiliations:** 1 Department of Biomedical Sciences, Centre National de Recherche et de Formation sur le Paludisme, Ouagadougou, Burkina Faso; 2 Department of Medical Microbiology, Radboud University Medical Centre, Nijmegen, The Netherlands; 3 Department of Infectious & Tropical Diseases, London School of Hygiene & Tropical Medicine, London, United Kingdom; 4 Centre for Immunity, Infection and Evolution, University of Edinburgh, Edinburgh, United Kingdom; 5 Department of Public Health, Erasmus MC, University Medical Centre Rotterdam, Rotterdam, The Netherlands; Sabin Vaccine Institute, United States of America

## Abstract

**Background:**

Man to mosquito transmission of malaria depends on the presence of the sexual stage parasites, gametocytes, that often circulate at low densities. Gametocyte densities below the microscopical threshold of detection may be sufficient to infect mosquitoes but the importance of submicroscopical gametocyte carriage in different transmission settings is unknown.

**Methodology/Principal Findings:**

Membrane feeding experiments were carried out on 80 children below 14 years of age at the end of the wet season in an area of seasonal malaria transmission in Burkina Faso. Gametocytes were quantified by microscopy and by *Pfs25*-based quantitative nucleic acid sequence-based amplification assay (QT-NASBA). The children's infectiousness was determined by membrane feeding experiments in which a venous blood sample was offered to locally reared *Anopheles* mosquitoes. Gametocytes were detected in 30.0% (24/80) of the children by microscopy compared to 91.6% (65/71) by QT-NASBA (p<0.001). We observed a strong association between QT-NASBA gametocyte density and infection rates (p = 0.007). Children with microscopically detectable gametocytes were more likely to be infectious (68.2% compared to 31.7% of carriers of submicroscopical gametocytes, p = 0.001), and on average infected more mosquitoes (13.2% compared to 2.3%, p<0.001). However, because of the high prevalence of submicroscopical gametocyte carriage in the study population, carriers of sub-microscopical gametocytes were responsible for 24.2% of the malaria transmission in this population.

**Conclusions/Significance:**

Submicroscopical gametocyte carriage is common in an area of seasonal transmission in Burkina Faso and contributes substantially to the human infectious reservoir. Submicroscopical gametocyte carriage should therefore be considered when implementing interventions that aim to reduce malaria transmission.

## Introduction

The transmission of malaria depends on the presence of mature sexual stage parasites, gametocytes, in the human peripheral blood. Once ingested by a mosquito taking a blood meal, gametocytes develop through different mosquito-specific stages and ultimately result in infection of the mosquito salivary glands with sporozoites. This renders the mosquito infectious to humans. There is growing awareness that targeting gametocytes, either alone or as part of integrated control programmes, is essential for malaria control and elimination efforts [1], [2], [3], [4]. The identification of the human infectious reservoir is therefore important for successful malaria control. Gametocytes frequently occur at low densities, making microscopical detection complicated [5]. In the last decade, molecular tools have become available to detect and quantify gametocytes at densities well below the microscopical threshold, in the order of 0.02–10 gametocytes/µL of blood [4]. Using these techniques, it has become evident that the proportion of gametocyte carriers in the population has been grossly underestimated and that the gametocyte reservoir may be 2–5 fold larger than assumed based on microscopy [6], [7]. Carriers of gametocytes at submicroscopical levels are capable of infecting mosquitoes [7], [8], [9], [10], [11], [12], [13], [14], [15], [16], [17], [18], [19] although at a lower degree than those with gametocytes detectable microscopically in whom gametocytes are present at higher densities [12], [18].

The importance of submicroscopical gametocyte carriage for malaria epidemiology and malaria control is the subject of some debate. While carriers of gametocytes at submicroscopical densities were concluded to be as important for the human infectious reservoir as carriers of microscopically-detectable gametocytes in areas of perennial transmission in Kenya [18] and Thailand [16], data from the Gambia suggest that submicroscopical gametocyte carriers only form a very small fraction of the infectious reservoir in this area of seasonal transmission [20]. This suggests that the relevance of submicroscopical gametocyte carriage may depend on transmission settings.

Here, we determine the contribution of submicroscopical densities of gametocytes to the human infectious reservoir in an area of seasonal malaria transmission in Burkina Faso.

## Methods

This study was conducted in September-November 2005 in the village of Laye, 30 km northwest of Ouagadougou, Burkina Faso. The area is characterised by Sudanese savannah with a marked wet season from June to October and an estimated entomological inoculation rate of 300–500 infective bites per person per year [21]. Asexual parasite carriage in the population shows seasonal fluctuations and was recently estimated at 60–90% in children below 15 years of age and 20–50% in adults [22]. Clearance was received by the Ministry of Health of Burkina Faso. Children below 14 years of age were randomly selected from village census lists and written informed consent was obtained from parents/guardians after the purpose of the study was explained. Children were accompanied to the Centre National de Recherche et de Formation sur le Paludisme (CNRFP) 1 to 2 days after the consenting procedure. At CNRFP, children underwent a clinical examination and their axillary temperature was measured. Children were enrolled in membrane feeding experiments regardless of symptoms or the presence of asexual malaria parasites or gametocytes. Venous blood samples (3 mL) were drawn into heparin-containing tubes for membrane feeding and for gametocyte detection both by microscopy and by real-time *Pfs25* quantitative nucleic acid sequence based amplification (QT-NASBA). For all membrane-feeding assays, 3 mL venous blood samples were obtained and fed to ∼50 locally colony-reared 4–5-day-old female *A. gambiae* sensu stricto mosquitoes. The mosquito colony was established three years prior to the current experiments. Blood was offered via an artificial membrane attached to a water-jacketed glass feeder maintained at 37°C. After 10–15 min, fully fed mosquitoes were selected and kept on glucose at 29°C [7]. Unfed and partially fed mosquitoes were removed by aspiration and discarded. Mosquito midguts were examined after 7 days for the presence of oocysts following dissection in 2% mercurochrome. A second microscopist confirmed the presence of oocysts in midguts that were scored positive.

Only the total number of oocysts per batch of fed mosquitoes was recorded; not the number of oocysts per individual mosquito.Experiments in which a minimum of 10 mosquitoes were examined on day 7 after feeding were included in the analyses. After membrane feedings, children with fever (axillary temperature ≥37.5°C) and malaria parasites were treated with artemisinin-based combination therapy according to the national guidelines. Individuals for whom infections other than malaria were suspected were accompanied to the nearest health facility for appropriate clinical care. The study protocol was viewed and approved by the Ministry of Health of Burkina Faso on August 8th 2000(Research's Authorization number 2000/3174/MS/SG/DEP).

### Microscopical Detection of *P. falciparum* Parasites

Samples were considered negative if no parasites were detected in 100 fields. Both asexual stage and gametocyte densities were simultaneously assessed by counting against 1000 leucocytes in the thick smear. The lower limit of microscopy for gametocyte quantification was therefore estimated at 8 gametocytes/ µl of blood. Parasite counts were converted to numbers of parasites per µl by assuming a standard count of 8000 leucocytes/µl of blood. Each sample was read independently by two microscopists and the mean density was used. A third reader was involved when the first two readers disagreed about the prevalence of gametocytes or their estimated densities differed ≥30%. In these cases the mean density of the two closest readings was used.

### Gametocyte Detection by Real Time Pfs25 QT-NASBA

Gametocyte detection by *Pfs25* QT-NASBA was performed as described elsewhere using a NucliSens EasyQ analyser (Bio-Mérieux) [23], [24]. Nucleic acid was extracted from 50-µL blood samples as described by Boom *et al.* [25]. The first part of the RNA extraction was done in the field following the original guanidinium isothiocyanate (GuSCN) RNA extraction method [25] until the nucleic acids were bound to silica dioxide particles. At this point, samples were stored at −20°C and transferred to the laboratory for completion of the extraction and QT-NASBA analysis. The number of gametocytes was calculated in relation to a standard gametocyte stage V dilution series [26], using the time point of amplification at which the fluorescence detecting target amplicons exceeded the mean fluorescence of three negative controls + 20 standard deviations. The *Pfs*25 QT-NASBA technique is gametocyte specific and has a detection limit of 20–100 gametocytes/mL[24]. Samples with *Pfs*25 QT-NASBA gametocyte concentrations <20 gametocytes/mL were considered gametocyte negative.

### Sample Size Considerations

Based on a previous study in the area, we expected a gametocyte prevalence of 10–20% by microscopy and 70–80% by *Pfs*25 QT-NASBA [23]. Including 80 individuals in the membrane feed experiments would allow us to detect a threefold lower infectiousness of submicroscopical carriers compared to microscopical gametocyte carriers [12], [20] when we assumed that 60% of the microscopical gametocyte carriers infected at least one mosquito [18] (Z_α_ = 1.645; Z_β_ = 0.84).

### Data Analysis

Data analyses were performed using SPSS version 16.0 (SPSS Inc., Chicago, IL, USA) and Stata 10 (Statacorp, Texas US). Densities of gametocytes were analysed on a log10-scale. The prevalence of mosquito infection (i.e. whether an individual infected at least one mosquito) and the proportion of infected mosquitoes were used as outcomes of the membrane feeding experiments and were related to *Pfs*25 QT-NASBA gametocyte density, age and fever in logistic and linear regression models. Individual oocyst densities in mosquitoes were not recorded.

## Results

**Figure 1:**
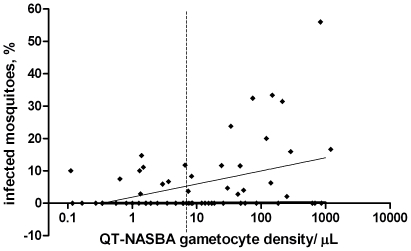
The relationship between *Pfs*25 QT-NASBA gametocyte density and the proportion of infected mosquitoes in membrane feeding experiments. The solid line indicates the best fitted line (Y  =  0.0176Ln(X) + 0.0187; R^2^ = 0.153). The dashed line indicates the estimated microscopic threshold for gametocyte detection, 8 gametocytes/µL, when screening 100 high power fields (i.e. ∼1000 white blood cells).

We enrolled 80 children in our study who were aged 2.9–13.6 years. In line with previous studies from the study area, microscopy indicated an asexual parasite prevalence of 82.5% (66/80) and a gametocyte prevalence of 30.0% (24/80; Table 1) [22], [23]. When the *Pfs25* QT-NASBA was used for gametocyte detection, 91.6% (65/71) individuals were shown to be carrying gametocytes. There was a strong correlation between gametocyte densities detected by QT-NASBA and microscopy for microscopically gametocyte positive samples (Spearman correlation coefficient  =  0.60; p = 0.004). For nine individuals RNA collection failed, i.e. no sample was collected, and therefore no QT-NASBA data were available. Membrane feeds were successful for all 80 individuals but, due to mosquito mortality between the day of feeding and the day of dissection, only a total of 74 membrane feeds had at least 10 mosquitoes dissected and were therefore included in the analyses. The proportion of infected mosquitoes was positively associated with *Pfs*25 QT-NASBA gametocyte density (Spearman correlation coefficient = 0.34, p = 0.007; Figure 1) and was not influenced by a clinical malaria episode (i.e. fever with a parasite density ≥500 parasites/µL) (p = 0.18) or the presence of fever (p = 0.63).The relation between the proportion of infected mosquitoes and *Pfs*25 QT-NASBA gametocyte density was best described by the equation Y  =  0.0176Ln(X) + 0.0187 (R^2^ = 0.153).

**Table 1:** Baseline characteristics.

Age, median (IQR)	6.2 (4.8–9.5)
Sex, % male (n/N)	55.0 (44/80)
Fever, % (n/N)*	22.5 (18/80)
Asexual parasite prevalence, % (n/N)	82.5 (66/80)
Asexual parasite density/µL, median (IQR)¥	1028.5 (585–3662)
Symptomatic malaria¶	15.0 (12/80)
Microscopic gametocyte prevalence, % (n/N)	30.0 (24/80)
Microscopic gametocyte density/µL, median (IQR)¥	40.0 (16–45)
QT-NASBA gametocyte prevalence, % (n/N)†	91.6 (65/71)
QT-NASBA gametocyte density/µL, median (IQR)	7.9 (1.4–48.9)

* Fever = temperature ≥37.5°C;

¥ for carriers only;

¶ defined as fever with a parasite density ≥500 parasites/µL;

† only gametocyte densities ≥20 gametocytes/mL were considered *Pfs*25 QT-NASBA positive.

Of those children with microscopically detectable gametocytes, 68.2% (15/22) infected at least one mosquito compared to 31.7% (13/41) of children with submicroscopical gametocyte densities (p = 0.001; Table 2). The proportion of infected mosquitoes was also higher in carriers of microscopically- compared to submicroscopically-detectable gametocytes (p<0.001). Oocyst densities of individual mosquitoes were not recorded, only the total number of oocysts observed in each experiment. Formal statistical testing on oocyst burden could therefore not be done although the total number of oocysts relative to the number of infected mosquitoes was higher for individuals with microscopically-detectable densities of gametocytes. The relative contribution to transmission of microscopical and submicroscopical gametocyte carriage was determined based on their prevalence in the studied population and the proportion of mosquitoes infected by each group. This resulted in a relative contribution to malaria transmission of 24.2% for submicroscopical gametocyte carriage, compared to 75.8% for microscopical gametocyte carriage.

**Table 2:** Membrane feeding results.

	Gametocyte carriage				
	Microscopy –	Microscopy –	Microscopy +	Total	p-value
	QT-NASBA –	QT-NASBA +	QT-NASBA +		
Prevalence in the population, % (n)	6.9 (5)	60.3 (44)	32.9 (24)	100 (73)	
Density at feeding, median (IQR)*	N.D.	7.5 (1.5–27.9)	33.8 (2.9–120.2)	8.2 (1.6–49.7)	0.13
Proportion of infectious individuals, % (n/N)	0.0 (0/5)	31.7 (13/41)	68.2 (15/22)	41.1 (28/68)	0.001¶
Proportion of infected mosquitoes, % (n/N)	0.0 (0/151)	2.3 (28/1202)	13.2 (90/683)	5.8 (118/2036)	<0.001¶
Total number of oocysts/infected mosquitoes¥	0/0	36/28	250/90	286/118	
Relative contribution to transmission	0%	24.2%	75.8%	100.0%	

* by Pfs25 QT-NASBA;

¶ p-value for a test for trend;

¥ only the total number of oocysts per batch of fed mosquitoes was recorded, not the number of oocysts of individual mosquitoes. Therefore only a summary measure can be presented and no analyses could be done on individual oocyst densities.

The total number of samples is lower than 80 because QT-NASBA results were not available for 9 individuals. Two individuals without QT-NASBA results that were gametocyte positive by microscopy were included. The relative contribution to transmission was based on the product of the proportion of infected mosquitoes (4^th^ row) and the prevalence of this subgroup in the population (1^st^ row).

## Discussion

In this study, we observed that submicroscopical gametocyte carriage is common in children in an area of seasonal malaria in Burkina Faso. Although, on average, carriers of gametocytes at submicroscopical densities infected significantly fewer mosquitoes that themselves developed lower oocyst burdens, the contribution to the infectious reservoir of this age group was considerable and estimated at 24%.

There are numerous reports that suggest that some individuals can infect mosquitoes despite the absence of microscopically detectable gametocytes [7], [8], [10], [12], [16], [18], [19]. However, it is unclear (i) how important this phenomenon is in areas of seasonal malaria [20] and (ii) how important submicroscopical gametocyte densities are for malaria transmission in the general, typically asymptomatic, population. Several detailed studies on the infectiousness of carriers of submicroscopical gametocytes have been carried out, but only after chemotherapeutic treatment of symptomatic malaria cases [7], [18], [20]. Because children in those studies all had high densities of asexual parasites in the weeks prior to the mosquito feeding experiments, they were more likely to have gametocytes at the time of the membrane feedings [5]. Although it was previously shown that gametocyte carriage may be common in asymptomatic individuals as well [27], findings from clinical trials cannot be extrapolated to the general population. To the best of our knowledge, the infectious reservoir has never been determined in the general population by means of the combination of molecular gametocyte detection tools with membrane feeding experiments used in the study described here. Although our experiments were restricted to children, our findings provide valuable information about the infectiousness of a cross-section of the population living in an area of seasonal malaria transmission. At the end of the wet season, 90% of the children in our study area carried gametocytes [23], two-thirds of them at densities below the microscopical threshold of detection. The infectiousness of individuals with submicroscopical gametocyte densities was lower than that of children with microscopical gametocyte densities in terms of (i) prevalence of infection, (ii) proportion of infected mosquitoes and (iii) oocyst burden in mosquitoes. These observations are largely in agreement with previous studies [12], [18] although a study in symptomatic children in Kenya reported that submicroscopical gametocyte carriers were as likely as microscopical gametocyte carriers to be infectious and only the average number of infected mosquitoes and the oocyst burden were lower for submicroscopical gametocyte carriers [18]. This difference may be due to the different populations: we enrolled asymptomatic children compared to the Kenyan study where children were sampled 14 days after a clinical malaria episode [18]. We nevertheless consider the current findings biologically more plausible since the chance of a submicroscopical gametocyte carrier being classified as ‘infectious’ (i.e. infecting at least one mosquito) is likely to be lower if submicroscopical gametocyte carriers on average infect a lower proportion of mosquitoes [12], [18]. Contrary to a recently published hypothesis that infection of mosquitoes by submicroscopical gametocytaemia may be rare in areas of seasonal malaria transmission [20], our findings suggest that carriers of submicroscopical gametocyte densities may be common in these circumstances. The relative contribution to transmission per gametocyte carrier may be lower for submicroscopical gametocyte carriers but their relative abundance in a population appears to counterbalance this and makes them important contributors to malaria transmission. To reliably determine the influence of transmission intensity and seasonality on the occurrence and infectiousness of submicroscopical gametocyte densities, a direct comparison is needed where the infectiousness of different populations is assessed at several time-points during the season.

Our study has two limitations: we determined the infectious reservoir at the end of the wet season only and restricted our experiments to children. Seasonal patterns in gametocyte carriage [22] make it impossible to draw conclusions about the importance of submicroscopical gametocyte carriage for malaria transmission at other time-points in the season. For this, a series of membrane feeding experiments are needed throughout the year. We have previously reported that submicroscopical gametocyte carriage is less prevalent in adults in our study area [23]. Our data can therefore not be extrapolated to the whole population. Although we observed a significant correlation between *Pfs*25 QT-NASBA gametocyte density and mosquito infection rates, it is not possible to reliably estimate the infectiousness of individuals based on gametocyte density data only. Some children with a gametocyte density below 1 gametocyte/µL were able to infect mosquitoes in our study. This is surprising since a blood meal, that is on average 2–3µL, should contain at least one male and one female gametocyte to result in infection. However, the phenomenon has been observed before [18] and may be influenced by the aggregation of gametocytes that favours the encounter of males and females [28]. Alternatively, we cannot rule out that artefacts resulting from RNA degradation have resulted in unrealistically low estimates of gametocyte densities in occasional samples. We also observed that some carriers of gametocytes at high density were unable to infect mosquitoes [8], [12], [18]. This could be partly due to transmission reducing immune responses [8], [29]. These immune responses may be inversely related to age [18], [29]. Further studies should therefore be conducted at different time-points, include all age groups and preferably incorporate transmission reducing immune responses and sexing of gametocytes to further elucidate the detailed processes that determine the composition of the human infectious reservoir of malaria in a given transmission setting.
